# Early Results of Clinical Application of Autologous Whole Bone Marrow Stem Cell Transplantation for Critical Limb Ischemia with Buerger’s Disease

**DOI:** 10.1038/srep19690

**Published:** 2016-01-21

**Authors:** Seon-Hee Heo, Yoong-Seok Park, Eun-Suk Kang, Kwang-Bo Park, Young-Soo Do, Kyung-Sun Kang, Dong-Ik Kim

**Affiliations:** 1 Division of Vascular Surgery, Samsung Medical Center, Sungkyunkwan University School of Medicine, Seoul, Korea; 2 Department of Laboratory Medicine and Genetics, Samsung Medical Center, Sungkyunkwan University School of Medicine,, Seoul, Korea; 3 Department of Radiology and Center for Imaging Science, Samsung Medical Center, Sungkyunkwan University School of Medicine, Seoul, Korea; 4 Department of Veterinary Public Health, Seoul National University, Seoul, Korea

## Abstract

Our goal was to evaluate early results of the clinical application of autologous whole bone marrow stem cell transplantation (AWBMSCT) for critical limb ischemia (CLI) in patients with Buerger’s disease. We retrospectively analyzed the data of 58 limbs of 37 patients (mean age, 43.0 years; range, 28–63 years; male, 91.9%) with Buerger’s disease with CLI who were treated with AWBMSCT from March 2013 to December 2014. We analyzed Rutherford category, pain score, pain-free walking time (PFWT), total walking time (TWT), ankle brachial pressure index (ABPI), and toe brachial pressure index (TBPI), and investigated wound healing and occurrence of unplanned amputations. The mean follow-up duration was 11.9 ± 7.2 months (range, 0.9–23.9 months) and 100%, 72.4%, and 74.1% of patients were available to follow-up 1, 3 and 6 months after AWBMST, respectively. At 6 months, patients demonstrated significant improvements in Rutherford category (P < 0.0001), pain score (P < 0.0001), PFWT (P < 0.0001) and TBPI (P < 0.0001). ABPI was increased compared to baseline, but the difference was not significant. A total of 76.5% ischemic wounds achieved complete or improved healing. AWBMSCT is a safe and effective alternative or adjunctive treatment modality to achieve clinical improvement in patients with CLI.

The prognosis of patients with critical limb ischemia (CLI), which represents ischemic rest pain and/or skin ulceration/gangrene^1^, is quite poor in terms of both survival and limb salvage despite conventional therapeutic options such as medications, surgical treatments and endovascular interventions^2^. Patients with Buerger’s disease, which is an inflammatory vascular disease affecting small- and medium-sized vessels characterized by segmental thrombotic occlusions^3^, are treated using surgical and endovascular methods that have limitations including relatively low patency rates and choice of target artery. A number of medical treatments for CLI have been studied, but none has obtained universal acceptance except for smoking cessation^4^. Therefore, new treatment strategies for revascularization have been developed and the use of therapeutic angiogenesis using stem cell transplantation has been studied to treat patients for whom surgical or radiologic intervention is neither successful nor indicated. Several studies of stem-cell based therapeutic angiogenesis have shown that stem cell-based therapeutic angiogenesis is a safe, feasible and potentially effective treatment option^5^,^6^,^7^. Recently, studies of bone marrow aspirate injection for CLI showed that bone marrow cell therapy is a potential option in CLI patients who are not candidates for bypass or endovascular intervention^8^.

Autologous whole bone marrow stem cell transplantation (AWBMSCT) has been approved as a common treatment in patients with CLI and denoted a new health technology by the Ministry of Health and Welfare (South Korea) in 2013. The indication for AWBMSCT in patients with CLI is Rutherford category of 4–6^2^,^6^,^7^,^9^. The aim of this study was to evaluate early results of clinical applications of AWBMSCT for the treatment of CLI in patients with Buerger’s disease.

## Results

From March 2013 to December 2014, of 58 limbs of 37 patients (mean age, 43.0 years; range, 28–63 years; male, 91.9%) with critical limb ischemia were treated with AWBMSCT. Baseline characteristics and previous medical and surgical treatment histories of the patients were shown in Table 1. Among 58 limbs, 20 limbs were treated with previous stem cell therapy and the mean treatment interval period was 6.8 ± 2.3 years (range, 4.0–10.4 years). The same surgical team performed all procedures. The average numbers of total mononuclear cells (MNC) and CD34 + cells in the peripheral blood and bone marrow are shown in Table 2. In each injection, a mean of 1.93 × 10^7^ mononuclear cells were delivered in a volume of 1 ml.

No patients experienced procedure-related complications such as bleeding, hematoma formation, injection site infection, osteomyelitis, or major systemic complications such as myocardial infarction or stroke during the hospital stay.

### Treatment Results

The mean follow-up duration after AWBMST was 11.9 ± 7.2 months (range, 0.9–23.9 months) and 58 limbs (100%), 42 limbs (72.4%) and 43 limbs (74.1%) were available to follow-up 1, 3 and 6 months after AWBMST, respectively.

**Figure 1:**
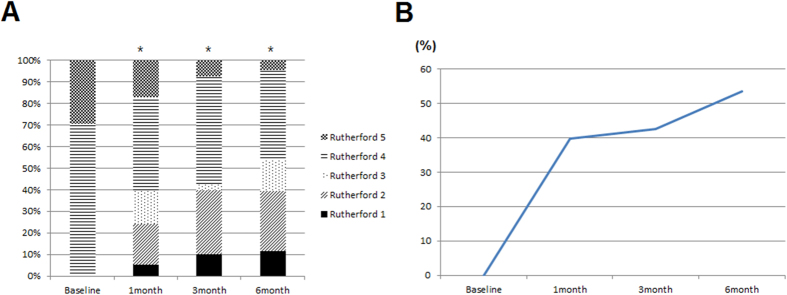
Serial changes in Rutherford category (0–6) (**A**) and CLI-free ratio (**B**) after AWBMSCT (n = 58 at 1 month, n = 42 at 3 months, n = 43 at 6 months). *Represents P < 0.05 compared with baseline data.

Among 58 limbs, the Rutherford category of 4 was in 41 limbs (70.7%) and 5 was in 17 limbs (29.3%) at baseline. After AWBMSCT, the Rutherford category significantly improved by 1 month and this improvement was sustained at 6 months (Fig. 1A). The proportion of the patients in the Rutherford category of 0–3 (CLI – free ratio) serially increased (Fig. 1B). These results suggest that AWBMSCT achieved clinical improvement and may lead to significant retrieval from CLI.

**Figure 2:**
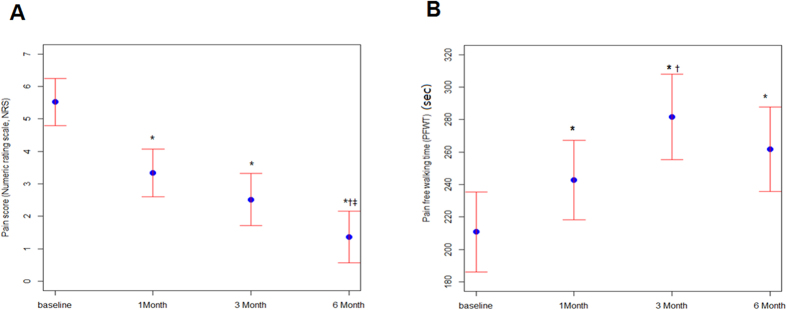
Serial changes in pain score (numeric rating scale, NRS) (**A**) and pain-free walking time (PFWT)(sec) (**B**) after AWBMSCT (n = 58 at 1 month, n = 42 at 3 months, n = 43 at 6 months). *Represents P < 0.05 compared with data at baseline, ^†^Represents P < 0.05 compared with data at 1 month after treatment, ^‡^Represents P < 0.05 compared with data at 3 months after treatment.

To evaluate the improvement of “symptomatic relief”^10^, we analyzed pain scores (NRS), PFWT and TWT. The mean pain score was 5.5 ± 0.4. Pain scores were significantly decreased at 1 month (3.3 ± 0.4, P < 0.001 vs. baseline), 3 months (2.5 ± 0.4, P < 0.001 vs. baseline) and 6 months (1.4 ± 0.4, P < 0.001 vs. baseline) (Fig. 2A). PFWT was significantly increased at 1 month (243 ± 12 sec, P = 0.0028 vs. baseline), 3 months (282 ± 13 sec, P < 0.001 vs. baseline) and 6 months (262 ± 13 sec, P < 0.001 vs. baseline) (Fig. 2B). These outcomes indicate improvements in the quality of life of patients.

**Figure 3:**
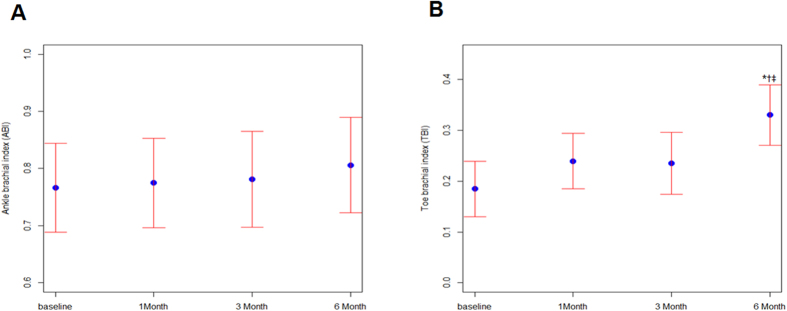
Serial changes in ankle brachial pressure index (ABPI) (**A**) and toe brachial pressure index (TBPI) (**B**) after AWBMSCT (n = 58 at 1 month, n = 42 at 3 months, n = 43 at 6 months). *Represents P < 0.05 compared with data at baseline, ^†^Represents P < 0.05 compared with data at 1 month after treatment, ^‡^Represents P < 0.05 compared with data at 3 months after treatment.

ABPI was increased from 0.76 ± 0.03 at baseline to 0.78 ± 0.04 after 3 months and to 0.81 ± 0.04 after 6 months, but the difference was not significant (Fig. 3A) However, TBPI was significantly improved from 0.18 ± 0.03 at baseline to 0.33 ± 0.03 after 6 months (P < 0.0001) (Fig. 3B).

Among 46 limbs, 17 limbs had an ischemic ulcer. The ischemic ulcers completely healed in 11 limbs (64.7%) and improved markedly in 2 limbs (11.7%) after 6 months. During the follow-up period, the ischemic ulcer of 4 limbs (23.5%) did not change and there was only 1 unplanned toe amputation due to severe pain. There were no newly developed cases of ischemic ulcers after treatment.

## Discussion

Critical limb ischemia (CLI) is the most advanced stage of peripheral arterial disease and is characterized by ischemic rest pain or tissue loss^8^. In the past decade, therapeutic angiogenesis has been studied for the treatment of patients with CLI who are not candidates for surgical or interventional treatments^11^,^12^. Tateishi *et al.* published the first pilot study and randomized controlled trial (RCT) indicating that autologous BM-derived cell therapy could be a safe and effective option for the achievement of therapeutic neovascularization in CLI^13^. Other small, noncontrolled, and nonblinded case series have reported positive effects of cell therapy on both subjective and objective surrogate endpoints, such as pain scores, pain-free walking distance, ankle-brachial index and transcutaneous oxygen measurements^12^,^14^,^15^. A meta-analysis published in 2013 also showed the beneficial effects of BM-derived cell therapy^8^. Moreover, RCTs showed reduced amputation rates with relative risks of major amputation^16^,^17^,^18^.

The main reason for the improvement in CLI clinical status is thought to be the angiogenic effect of the injected stem cells. We reported that angiogenic vessels developed after stem cell therapy in a 2006 clinical study^6^. In addition to the angiogenic effects of stem cells, the current theory was more focused on cytokine effects related to stem cells.

Our sample was heterogeneous regarding smoking cessation status, but only about 17% of patients had quit smoking 3 months before treatment and the smoking status of most patients (83%) did not change. The majority of patients exhibited improvements of clinical status, so we hypothesized that clinical improvement was due to angiogenic effects rather than change of smoking status.

We used autologous whole bone marrow stem cells for therapeutic angiogenesis in our study, but other recently published studies on therapeutic angiogenesis have used bone marrow mononuclear cells (BM-MNCs) or peripheral blood-derived mononuclear cells (PB-MNCs)^19^,^20^. However, the isolation of MNCs from whole bone marrow cells or peripheral blood cells is highly complex and expensive and is susceptible to contamination^6^. Moreover, many cytokines in whole bone marrow stem cells can be delivered effectively without manipulation.

Recently, our institution published the results of a clinical trial of AWBMSCT^6^,^7^. AWBMSCT has since been adopted as a new health technology by the Ministry of Health and Welfare (South Korea) and we have regularly performed AWBMSCT on patients with critical limb ischemia as of March 2013. This study represents the first published results after the adoption of AWBMSCT as a new health technology in South Korea.

Measurements of transcutaneous O2/CO2 (tcpO2/tcpCO2) are very useful as markers of microvascular assessment after stem cell therapy^21^. Unfortunately, we were unable to measure tcpO2/tcpCO2 due to a lack of equipment in our hospital.

In this study we included only a small number of patients and presents only the early results of AWBMSCT. Because this is retrospective study, we were unable to control for smoking cessation status. Further long-term follow up studies are needed to validate the clinical effects of AWBMSCT using a control group to compare clinical status and angiography.

Our results suggest that patients with critical limb ischemia due to Buerger’s disease achieve clinical improvement after AWBMSCT. We observed significant improvements of clinical status involving subjective as well as objective parameters such as TBPI and degree of wound healing. In particular, the improvements in the pain score and PFWT suggest symptomatic relief and improvements in the quality of life of patients. This study presents AWBMSCT as a relatively safe treatment modality without procedure-related or systemic complications. Therefore, AWBMSCT could be a safe, effective alternative treatment modality to achieve clinical improvement in patients with CLI refractory to other treatment modalities.

## Methods

### Patient population

From March 2013 to December 2014, 58 limbs of 37 patients of Buerger’s disease with CLI were treated with AWBMSCT in a single institution. We retrospectively collected demographic and clinical data for all patients. All patients were examined by lower extremity CT angiography for differential diagnosis and to assess the feasibility of bypass or endovascular treatment options. Only patients who were not candidates for bypass or endovascular intervention were selected for inclusion in the study. We did not include a control group to compare to patients received AWBMSCT.

The diagnosis of Buerger’s disease was based on Shinoya’s criteria^10^. Treatment indications were: (1) critical limb ischemia with a graded Rutherford category of 4–6^1^, (2) sustained ischemic symptoms for more than 3 months despite conservative medical treatment, (3) no indication of endovascular treatment or bypass surgery, and (4) age 20–80 years. Conservative medical treatment included smoking cessation, foot care, and medical treatment (antiplatelet agent, vasodilator, calcium channel blocker, cilostalzol). The choice of medical treatment was made according to patient condition and medical history.

### Treatment

Recombinant human granulocyte colony-stimulating factor (r-HuG-CSF; Leucostim^®^, Dong-A ST, Seoul, Korea) was injected subcutaneously daily at 6 p.m. for 5 days before stem cell transplantation. r-HuG-CSF doses were adjusted daily according to leukocyte numbers in the peripheral blood. r-HuG-CSF was injected subcutaneously at 75 μg daily until leukocyte numbers reached 20,000 cells/ml, but was reduced to 50 μg when leukocyte numbers exceeded this figure. In the prone position under spinal anesthesia, approximately 40 ml of autologous whole bone marrow stem cells were aspirated via puncture with a bone marrow needle through the ipsilateral posterior superior iliac spine. The collected whole bone marrow (30 ml) was immediately split into 1-ml aliquots using 2-mm syringes. One milliliter of whole bone marrow stem cells was implanted into 30 points on the calf muscles in the vicinity of the native anterior and posterior tibial and peroneal arteries. The procedures were finished after placing compression dressings on the injection sites.

### Assessment of clinical status and follow-up

On admission, all patients underwent physical examinations and laboratory tests including complete blood count with differential count, liver function test, renal function test, fasting blood glucose, urine analysis, C-reactive protein, prothrombin time and activated partial prothrombin time, chest x-ray, and electrocardiogram. Medical experts assessed the presence of a wound with ulcers/gangrene on the extremity and recorded its location, size and depth. Patients underwent noninvasive vascular examinations including segmental limb pressure measurements on the treadmill (speed of 2 mph, 176 ft/min, maximum 5 minutes). Ankle brachial pressure index (ABPI), toe brachial pressure index (TBPI), pain-free walking time (PFWT), and total walking time (TWT) were also recoded. A pain score (numeric rating scale, NRS) was recorded by the patient for pain assessment. Physical examination and laboratory tests including complete blood count with differential count and C-reactive protein were performed to monitor for complications after treatment during hospital stay. All patients continued taking the same medication after AWBMSCT until follow-up.

To evaluate improvement of clinical status, patients came to the outpatient clinic 1, 3 and 6 months after treatment and underwent a physical examination and segmental limb pressure measurements on the treadmill and recorded their pain scores. During the follow-up period, unplanned amputations of the treated leg were recorded.

### Statistical analysis

We conducted a mixed model analysis with an autoregressive covariance structure to evaluate longitudinal variation of pain score, ABPI, TBPI, PFWT, and TWT, and serial change in the Rutherford category was analyzed using a generalized estimating equation. The differences between baseline and each follow-up examination after AWBMSCT were assessed using the Scheffe test while adjusting for age and treatment location (right limb or left limb). The significance level was set at 0.05 for all statistical tests. Statistical analyses were performed using SAS version 9.3 (SAS Institute, Cary, NC, USA) and R 3.0.1 (Vienna, Austria; http://www.R-project.org/).

Informed consent was obtained from all subjects. And this retrospective study was approved by the Institutional Review Board of the Samsung Medical Center and carried out in accordance with approved guidelines.

## Additional Information

**How to cite this article**: Heo, S.-H. *et al.* Early Results of Clinical Application of Autologous Whole Bone Marrow Stem Cell Transplantation for Critical Limb Ischemia with Buerger’s Disease. *Sci. Rep.* **6**, 19690; doi: 10.1038/srep19690 (2016).

## Figures and Tables

**Table 1:** Baseline clinical characteristics (N = 58 limbs for 37 patients).

Variable	No. (%)
Age (years, mean ± SD) (range)	43.0 ± 8.8 (28–63)
Gender, male	34(91.9)
Pain at rest	53(91.4)
Ischemic nonhealing ulcer	17(29.3)
Smoking	
Quit smoking ≥ 3 months prior to the treatment	33(56.9)
Quit smoking < 3 months prior to the treatment	10(17.2)
Current smoker	15(25.8)
Previous history of revascularization	
Bypass surgery	4(6.9)
Endovascular therapy	2(3.4)
Stem cell therapy	20(34.5)
Previous medical treatment	
Vasodilator	41(70.6)
Aspirin/clopidogrel	17(37.9)
Cilostazol	5(8.6)
Calcium channel blocker	10(17.2)

SD, standard deviation.

**Table 2:** Average number of total mononuclear cells (MNCs) and CD34 + cells in peripheral blood and bone marrow.

	Day	Total MNCs (/ml)	CD 34 + (/ml)
		Mean No.	Mean No.
PB	Preoperative day	3.10 × 10^6^ ± 0.75 × 10^6^	2.93 × 10^3^ ± 1.79 × 10^3^
	Op day	3.95 × 10^6^ ± 0.88 × 10^6^	1.20 × 10^4^ ± 3.58 × 10^4^
	Postoperative day	3.45 × 10^6^ ± 0.65 × 10^6^	
BM	Op day	1.93 × 10^7^ ± 1.36 × 10^7^	2.07 × 10^5^ ± 1.99 × 10^5^

MNCs, mononuclear cells; CD, cluster of differentiation; PB, peripheral blood; BM, bone marrow.
